# Interprofessional continuing education in health professions – a scoping review of framework conditions, design processes and evaluation designs

**DOI:** 10.3205/zma001815

**Published:** 2026-02-17

**Authors:** Bärbel Wesselborg, Ingo Knepperges, Nina von den Driesch, Miriam Schäfer, Astrid Stephan

**Affiliations:** 1 Fliedner Fachhochschule Düsseldorf, Studiengang Berufspädagogik Pflege und Gesundheit, Düsseldorf, Germany; 2 Uniklinik RWTH Aachen, Stabstelle Pflegewissenschaft der Pflegedirektion, Aachen, Germany

**Keywords:** interprofessional learning, methods, lecturers, evaluation

## Abstract

**Objective:**

Little is known about the design of interprofessional continuing education programs and the extent of the professional backgrounds of participants and lecturers, even though this information can be important for planning, implementing and achieving objectives. The aim of this study is therefore to provide a systematic overview of the framework conditions, design processes and evaluation designs of the interprofessional continuing education programs described in these studies.

**Methods:**

A scoping review was conducted. A systematic literature search was carried out in the MEDLINE, CINAHL, PROSPERO and ProQuest databases. In addition, relevant, nonindexed journals and grey literature were included and searched manually. The studies were selected on the basis of defined criteria and analysed deductively according to framework conditions and process and outcome criteria on the basis of Freeth and Reeves’ “3P model of learning to collaborate”.

**Results:**

Analysis of the included studies (n=79) revealed that interprofessional continuing education courses are used mainly by members of the medical and nursing professions. Some of the courses are led by interprofessional teams. The pedagogical qualifications of the instructors are rarely reported. Courses often take place in acute inpatient settings as part of emergency simulations. Furthermore, the care of people with certain diseases and communication are addressed relatively frequently. The continuing education courses are evaluated mainly by the participants.

**Conclusion:**

To assess the quality of interprofessional continuing education courses, the framework conditions should also be reported in full. Interprofessional events should increasingly offer cross-setting and cross-sector topics and include the perspective of patients in the evaluation.

## Zusammenfassung

**Zielsetzung:**

Weitgehend unbekannt ist, wie interprofessionelle Fortbildungen gestaltet werden und welche beruflichen Hintergründe Teilnehmenden und Dozierende mitbringen, obschon diese für die Planung, Durchführung und Zielerreichung wichtig sein können. Ziel der Studie ist es daher, einen systematischen Überblick über die in Studien beschriebenen Rahmenbedingungen, Gestaltungsprozesse und Evaluationsdesigns interprofessioneller Fortbildungsangebote zu geben.

**Methodik:**

Ein Scoping Review wurde durchgeführt. Die systematische Literaturrecherche wurde in den Datenbanken MEDLINE, CINAHL, PROSPERO und ProQuest durchgeführt. Zusätzlich wurden relevante, nicht indexierte Zeitschriften und graue Literatur einbezogen und manuell durchsucht. Die Auswahl der Studien erfolgte anhand definierter Kriterien und die Analyse erfolgte in Anlehnung an das „3P model of learning to collaborate“ von Freeth und Reeves deduktiv nach Rahmenbedingungen, Prozess- und Ergebniskriterien.

**Ergebnisse:**

Die Analyse der eingeschlossenen Studien (n=79) zeigt, dass interprofessionelle Fortbildungen mehrheitlich von Angehörigen der Medizin und Pflege genutzt werden. Die Fortbildungen werden teils von interprofessionellen Teams geleitet. Eine pädagogische Qualifikation der Dozierenden wird selten berichtet. Die Fortbildungen finden häufig im akutstationären Setting im Rahmen von Notfallsimulationen statt. Weiterhin wird häufiger die Versorgung von Menschen mit bestimmten Krankheiten sowie Kommunikation adressiert. Die Fortbildungen werden v.a. durch die Teilnehmenden evaluiert.

**Schlussfolgerung:**

Um die Qualität interprofessioneller Fortbildungen bewerten zu können, sollten auch die Rahmenbedingungen vollständig berichtet werden. Interprofessionelle Fortbildungsveranstaltungen sollten vermehrt setting- und sektorübergreifende Themen angeboten werden und die Perspektive von Patient:innen sollte in die Evaluation aufgenommen werden.

## 1. Introduction

Interprofessional education (IPE) is intended to play a central role in the qualification of health care professionals to improve interprofessional collaboration (IPC) in professional practice and thus health care [1]. In preparation for work scenarios implementing interprofessional teams, various educational programs have been developed worldwide in recent decades – with a time lag in German-speaking countries – to promote joint team-based learning (e.g., [2], [3], [4]).

In the context of interprofessional learning programs, formats have become established internationally that take place after initial qualification and are referred to as continuing interprofessional education (CIPE) [5], [6]. While IPE aims to improve the skills of students, CIPE focuses on professionals who are already working in various areas of the health care system. The terms IPE and CIPE are applicable only when members of two or more health care and/or social professions learn with, from and about each other to improve collaboration and the quality of health care [7]. There is evidence that IPE can improve the quality of care and patient outcomes both before and after professional registration [8], [9], [10].

Freeth and Reeves [6] describe fundamental didactic considerations regarding the structure and decision-making levels of interprofessional education programs. They adapted the 3P model (presage – process – product) for the design of learning situations of Biggs [11] to the specifics of interprofessional learning. In the “3P model of learning to collaborate” [6], important aspects of the planning and conducting of interprofessional learning formats, such as the specific educational outcomes that can be expected, are highlighted.

As interprofessional education in health professions has been the focus of attention and further development in the German-speaking countries of Germany, Switzerland, and Austria (GSA) in recent years, more interprofessional continuing education after entry into the profession is expected in the future [12]. Nevertheless, no studies have yet reported in detail the framework conditions (including the professional backgrounds of the participants and the profiles of the lecturers), didactic-methodological design decisions or evaluation designs. Consequently, this review aims to provide an overview of the conditions and didactic decisions in the methodological design and evaluation of interprofessional continuing education programs. In line with the assumed increase in the number of these courses [12], the results should have implications for the development of interprofessional continuing education programs, particularly in the GSA region.

The following research question was pursued:

- What framework conditions, didactic-methodological decisions and evaluation designs are described in the literature for interprofessional continuing education programs for health professions?

## 2. Method

### 2.1. Research design

A scoping review [13] was conducted to answer the research question. Scoping reviews are suitable for reviewing large amounts of (heterogeneous) literature and providing an overview of the research landscape. The Joanna Briggs Institute (JBI) framework for scoping reviews [14] was used for the review process.

### 2.2. Data collection: Search strategy

The PCC scheme (population, concept and context) was used to specify the research question and define the inclusion and exclusion criteria for the studies [14]. It was determined that the publications must have been related to professionals in the health care sector and social professions working in the health care sector (e.g., social work) [1], [7]. The courses must have taken place after professional certification to maintain or expand professional skills in the professional field and should not, in the sense of continuing education, have ended with an additional qualifying degree. In addition, the courses must have been evaluated. Furthermore, the continuing education should, by definition, have enabled interprofessional learning [7], i.e., used interactive teaching and learning methods that promoted exchange between professionals to increase interprofessional cooperation and the quality of care in the health care system.

The inclusion and exclusion criteria are presented in table 1 (Tab. 1).

A systematic literature search was conducted in the MEDLINE, CINAHL, PROSPERO and ProQuest databases on 25 February 2025 (see attachment 1 ). In addition, the relevant journals *“Journal of Research in Interprofessional Practice and Education”* and *“Health, Interprofessional Practice and Education”*, which are not listed in the databases, were searched manually.

The search string used included operationalised terms from the research questions and synonyms such as “interprofessional”, “interdisciplinary”, “interoccupation”, “team”, “continuing”, “postgraduate”, “ongoing”, “health care outcomes” and “educational outcomes”. The search included all English- and German-language articles. To obtain a broad overview of the research landscape, no filter was set with regard to the publication period.

**Figure 1:**
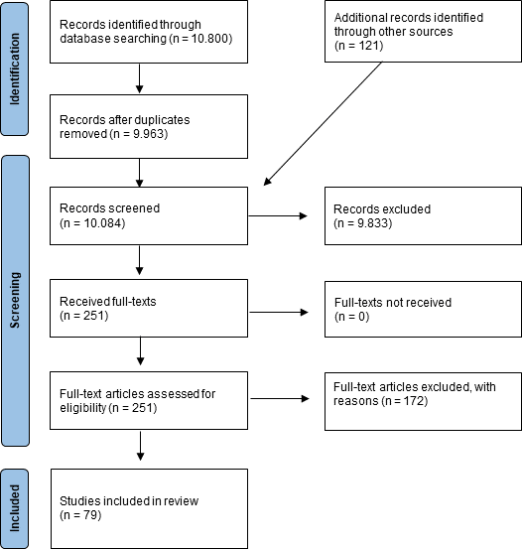
PRISMA flow chart (see [72])

A total of 10,800 studies were identified in the databases and 121 publications through manual searches. After title/abstract screening and exclusion of studies on the basis of the specified criteria (see table 1 (Tab. 1)), 79 studies were included (see figure 1 (Fig. 1)).

### 2.3. Data analysis

First, the basic bibliographic data of the included studies were extracted [15]. The studies were then analysed deductively on the basis of the “3P model of learning to collaborate” [6] using the categories “framework conditions” (presage), “didactic-methodological decisions” (process) and “evaluation” (product). The subcategories “context”, “lecturers” and “participants” were formed under framework conditions. To ensure the quality of the data extraction, 25 randomly selected studies (32%) were double-evaluated by the authors and showed a high degree of agreement. The remaining data were extracted by one person. The results were then analysed in terms of frequency and summarised.

**Figure 2:**
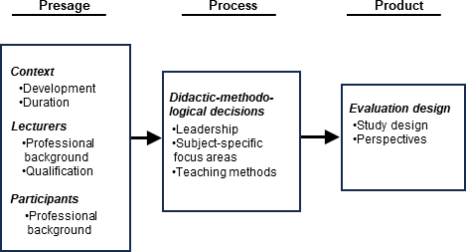
Evaluation categories based on the “3P model of learning to collaborate” [6]

All the evaluation categories are shown in the attached figure (see figure 2 (Fig. 2)).

Because a scoping review does not necessarily include an analysis of the study quality [16] and the study objective does not involve a methodological evaluation but merely a description of the included literature, the presentation of study quality was omitted.

## 3. Results

A total of 79 studies from 17 countries were included. Most of the studies originated from the USA (42%; n=33), Canada (18%; n=14), Great Britain (10%; n=8) and Australia (6%; n=5). Fewer studies on interprofessional continuing education were conducted in Sweden, the Netherlands and Austria, among other countries.

With the exception of one study [17], all continuing education courses were published after the turn of the millennium. Between 1996 and 2015, the number of publications almost doubled every five years.

The studies identified evaluated either team training for existing teams (e.g., [18]) or continuing education courses with participants from different fields of work (e.g., [19]). In addition, since the early 2020s, digitally supported programs offering national [20] and international [21] interprofessional continuing education courses have increased. In addition to studies that described the evaluation of a single course, studies that aimed to collect and evaluate data from several cohorts and courses were also included (e.g., [22]).

### 3.1. Framework conditions

#### 3.1.1. Context

##### 3.1.1.1. Development

The didactic concept of interprofessional continuing education is very often (66%; n=52) developed by the authors (e.g., [23], [24]). Only a few studies have justified the development of the program on the basis of the intended study objectives [17], [23], [25], [26], [27], [28], [29]. Standardised concepts such as “crew resource management” [30], “TeamSTEPPS” [31], “situation awareness” [32] or “serious illness conversation” [33] are cited as models for didactic structure. Furthermore, didactic concepts are developed on the basis of nationally implemented programs (e.g., [34]) or recommendations such as those of the Canadian patient safety institute (e.g., [35]).

##### 3.1.1.2. Duration

The duration of the course varied greatly. Most often, interprofessional courses lasted 2.5-5 hours (22%; n=17) or 6-8 hours (16%; n=13) and were conducted on a single day. However, the time frame was not reported in more than one-third of the studies (32%; n=25). One of the longest courses lasted 40 days [36] and focused on improving the general safety of patients in hospitals in just over 70 hours of theory and additional self-study and project work phases. At 2.5 hours each, the interprofessional courses offered by Abulebda et al. [37] and Bosnic-Anticevich et al. [25] were the shortest, each lasting 2.5 hours. These were a simulation-based team training course on paediatric emergencies [37] and a course on developing improved skills in educating patients with asthma on the use of metered-dose inhalers [25].

#### 3.1.2. Lecturers

##### 3.1.2.1. Professional background

The professional background of the lecturers is described in approximately half of the studies (47%; n=37). These were most frequently (usually in interprofessional teams; see didactic-methodological decisions) members of medicine (n=33) and nursing (n=27). Psychologists (n=8) were less frequently involved. Pharmacists (n=7) and physiotherapists (n=5) are mentioned somewhat less frequently. Occasionally, lecturers with professional backgrounds in medical technology, social work, midwifery, or nutritional science were involved.

##### 3.1.2.2. Qualification

The qualifications of lecturers for interprofessional continuing education courses are only reported in a fragmentary manner. In approximately half of the studies (n=37; 47%), no statement is made regarding qualifications. The most frequently mentioned qualification is a professional qualification in the subject area addressed (25%; n=20). Pedagogical qualifications are rare. In a few studies, lecturers are described as having a professional and (unspecified) pedagogical qualification (n=8). In some studies, further qualifications tailored to the specific training method (e.g., a Master's degree in Team-Stepps [38] or a debriefing course [37]) are mentioned for the lecturers (n=13). Adams et al. [39] emphasise that the lecturer is experienced in interprofessional education. The distinguishing features of this experience are not explained in detail.

#### 3.1.3. Participants

##### 3.1.3.1. Professional background

The included studies (based on reported case numbers) involved 11,273 members from 19 different health and social care professions. The most frequently involved professionals were medical doctors (100%; n=79) and nurses (90%; n=71). They were followed by pharmacists (32%; n=25), social workers (27%; n=21), and occupational therapists (23%; n=18) an physiotherapists (22%; n=17). In some studies, administrative or management staff were involved (13%; n=10), and in another study, pastoral carers were involved (6%; n=5). The most frequently involved professional groups are shown in table 2 (Tab. 2).

### 3.2. Process

#### 3.2.1. Didactic-methodological decisions

##### 3.2.1.1. Leadership

As described in the context section, information about the lecturers is provided in approximately half of the courses. In most cases (43%; n=34), teaching was carried out by an interprofessional team. Only one study does not describe this team in detail in terms of (professional) qualifications; otherwise, teams usually consisted of medical and nursing professionals and possibly others, such as social workers (e.g., [40]), physiotherapists (e.g., [41]) or pharmacists (e.g., [42]). Monoprofessional teams are rarely described (3%; n=2) [26], [43], and individual lecturers taught somewhat more frequently (10%; n=8) (e.g., [44]).

##### 3.2.1.2. Subject-specific focus areas

In interprofessional courses, emergency and resuscitation training (41%; n=32) in specific specialised areas in acute inpatient settings, such as gynaecology or intensive care medicine, and with existing teams are particularly frequently reported. These courses used self-developed or tried-and-tested programs (e.g., CRM or teamstep) and were designed to train technical and interprofessional skills to improve collaboration and health care (e.g., [32], [45]). In some cases, the courses also focused on teaching communication skills to improve interprofessional teamwork with little or no specialist content (9%; n=7) (e.g., [27], [40]).

Furthermore, continuing interprofessional education courses (28%; n=22) addressed the care of specific patient groups, e.g., those with diabetes [46] or psychiatric disorders [47]. These courses had a strong focus on specialist content and were located mostly in primary care. Specialist training courses that focused on different professional roles and perspectives on dealing with specific medications or medication management (5%; n=4) are also worth mentioning here (e.g., [48]). The remaining courses addressed topics such as communication training with difficult patients, patient safety and, in rare cases, transition and quality management (18% n=14) (e.g., [24], [39]).

##### 3.2.1.3. Teaching methods used

On average, four different methodological approaches were chosen in each interprofessional course. As described in the context section, the didactic concept of the course was very often (66%; n=52) developed by the authors. Only rarely (9%; n=7) was the development of the program justified on the basis of the study objectives. In addition to lectures (71%; n=56), which were used in the vast majority of interprofessional courses to impart knowledge, group discussions were the second most common (48%; n=38). Simulations with debriefings (49%; n=39) were also used. Other methods included case-based learning (38%; n=30), group work (14%; n=11) and practical exercises (25%; n=20). Role-playing (16%; n=13), self-regulated learning (19%; n=15) and educational films (14%; n=11) were also used. Since the early 2020s, video conference-based formats have been implemented (e.g., [21]).

### 3.3. Study data

#### 3.3.1. Evaluation design

##### 3.3.1.1. Study design

Approximately half of the studies (54%; n=43) had a quantitative study design. The remaining studies were based on a mixed-methods design (35%; n=28) or qualitative design (10%; n=8). The majority (68%; n=54) had at least two survey points (pre–post design), with only 6 cases being randomised controlled trials [32], [46], [49], [50], [51], [52].

##### 3.3.1.2. Evaluation perspectives

The studies almost always surveyed data from the participants’ perspective (96%; n=76). This was done by evaluating the interprofessional event itself, e.g., in terms of its organisation, or subjectively assessing relevance (e.g., [40], [53]). To evaluate interprofessional educational outcomes, reference was often made to the model developed by Barr and colleagues [54], originally based on Kirkpatrick’s model, which distinguishes between four levels: 1: reaction; 2a: change in attitude/perception; 2b: acquisition of knowledge and skills; 3: behavioural change; 4a: change in organisational practice; and 4b: benefit for the patient. Furthermore, changes in attitude or perception, e.g., towards team-based work, were surveyed from the participants’ perspective (e.g., [18], [55]). Many studies also investigated the acquisition of knowledge and skills, e.g., using questionnaires, sometimes before and after the course (pre-post design), e.g., perceived team performance or interprofessional collaboration and communication (e.g., [56]). However, there were also study designs that used knowledge tests to determine learning success before and after an event [57]. A few studies (8%; n=6) also investigated, beyond the participants' perspective, the extent to which changes occur in the organisational practice of health care after the course (e.g., [24], [38], [58]).

Furthermore, in some of the studies (16%; n=13), the perspectives of professional observers, particularly with regard to the implementation and assessment of the learning success of simulation-based emergency training (e.g., [37]), and, in one study, the statements of the simulation participants [49] were included in the evaluation.

Studies that aimed to demonstrate the benefits of the interprofessional course for patients (15%; n=12) generally used routine data analysis and presented, for example, changes (improvements) in patients' blood values or the (reduced) number of emergencies following interprofessional training (e.g., [51], [52], [59]). In one study [60], the benefits were also analysed via a focus group interview with patients.

## 4. Discussion

The scoping review aimed to produce an international overview of the framework conditions, didactic-methodological decisions and evaluations of interprofessional continuing education programs and to derive didactic implications, particularly for the GSA region. An analysis of the studies revealed that the subject matter of the courses often focused on team-based emergency and resuscitation training in an acute inpatient setting. Also the few studies from the GSA region focused primarily on team training in emergency situations (e.g., [61], [62]). This demonstrates the high demand, both internationally and in the GSA region, for interprofessional courses on how to address critical situations in the everyday work of health care professionals [63].

The majority of the studies focused on evaluating continuing education programs for team-based care of people with specific diseases. These continuing education programs focused on acquiring new knowledge and clarifying the roles and responsibilities of interprofessional teams. An important subject-specific impetus for possible national focal topics for interprofessional courses in the GSA region could come from health care research (including [https://www.aerzteblatt.de/themen/versorgungsforschung], accessed on 30 April 2025), which addresses the implementation of findings from basic medical research and clinical research in everyday practice. For example, as part of the implementation of the ‘health around birth’ action plan [64], continuing interprofessional education courses could be developed and offered to strengthen the intersectoral interprofessional cooperation outlined in the program. While simulation-based training courses in emergency scenarios were generally located in acute inpatient settings, events for patients with specific diseases often took place in primary care and outpatient health centres. However, few studies addressed cross-setting issues and transition management that link the individual health care sectors. The small number of studies with a cross-sectoral approach could be because, on one hand, unlike in Germany [65], the topic of transition management plays a weaker role in international settings, as established cross-sectoral care concepts exist in the respective national health care systems. On the other hand, it could be because setting- and thus institution-wide interprofessional courses involve a high level of organisational effort and are therefore rarely offered. This factor has already been highlighted as a barrier to interprofessional education in health care professions both internationally [66] and nationally [67]. In the GSA region, cross-setting courses should also be designed in the future against the backdrop of changing care requirements and short, acute inpatient stays [68]. In the future, educational institutions involved in continuing education for health care professionals should work together with health care researchers to design innovative interprofessional education courses, thereby improving patient care.

Notably, a total of 19 different professional groups were identified among the participants in the studies. However, the professional groups most frequently represented among both participants and lecturers were nursing and medicine. This reflects the situation in the GSA region, where these professions have the greatest number of employees in the German health care system ([https://de.statista.com/statistik/daten/studie/461487/umfrage/beschaeftigte-im-deutschen-gesundheitswesen-nach-arbeitsbereich/], accessed on 30 April 2025). However, new concepts should consider the extent to which other health care professionals can be involved in developing the objectives of continuing education, such as those in medical technology professions in the field of diagnostics [12], to address the topics more comprehensively on an interprofessional basis.

The studies contained little information about the pedagogical and didactic qualifications of the lecturers. It can be assumed that the lecturers are primarily qualified on the basis of their occupational expertise. Interprofessional education courses can be particularly challenging from a pedagogical point of view, as there may be prejudices and stereotypes towards other professional groups or reservations about changes, e.g., in the distribution of tasks in the health care system. Lecturers therefore need pedagogical skills that enable them to respond to unpredictable group dynamics and simultaneously create a positive learning environment that facilitates interaction between participants so that creative solutions to problems can be developed [6], [69]. Notably, many continuing education courses are led by interprofessional teams, which should also be the goal in the GSA region. This can help participants to better understand the different perspectives and roles of various health care professions and to try out interprofessional collaboration.

The evaluations generally incorporated the perspectives and subjective assessments of the participants. Only rarely were other perspectives, e.g., from observers during simulations, integrated. In some cases, routine data were used as surrogates for better quality of care. This is due to the difficulty of measuring interprofessional collaboration itself and its quality in clinical care, which is difficult and requires a great deal of effort. Studies very rarely systematically survey patients, even though they are the ultimate beneficiaries of interprofessional learning and collaboration. However, incorporating the subjective patient perspective could bring about a change in attitude among health care professionals, as is being promoted, for example, by the Swansea Bay University Health Board and the Patient Experience Network (PEN) ([https://patientexperiencenetwork.org/resources/case-studies/1793/], accessed on 30 April 2025) through the use of authentic case histories. Addressing the didactic principle of situation orientation is also in line with recommendations from continuing education regulations in health professions in the GSA region (including [70], [71]).

### 4.1. Limitations

This study has several limitations. First, the quality of the studies was not assessed, as the aim of the study was not to evaluate methodology but merely to describe the included literature. Furthermore, not all of the content of the studies was evaluated; instead, the focus was on a few characteristics of interprofessional content that had previously received little attention. With the exception of a sample for quality control, data extraction was carried out by only one person.

## 5. Conclusions

To ensure high-quality continuing education events, lecturers in interprofessional continuing education courses should have teaching qualifications. Continuing education courses should integrate other health professions more consistently and develop cross-setting offerings to respond to future needs. Technical topics could address current priorities in health care research and be carried out across institutions. The patient perspective should be included in the evaluation of such a course and as a didactic tool. The establishment of binding standards for reporting on studies of the development, implementation and evaluation of interprofessional education in the health care sector is desirable. Mandatory standards could also help lecturers reflect more deeply on didactic issues when designing continuing interprofessional education courses. In particular, to aid in the design of programs for promoting interprofessional skills, the standards could specify the methodological requirements for interprofessional learning to distinguish between multiprofessional and interprofessional learning [7], [69].

## Author’s ORCID

Bärbel Wesselborg: [0000-0001-8873-919X]

## Competing interests

The authors declare that they have no competing interests. 

## Supplementary Material

Search strings in the literature databases

## Figures and Tables

**Table 1:**
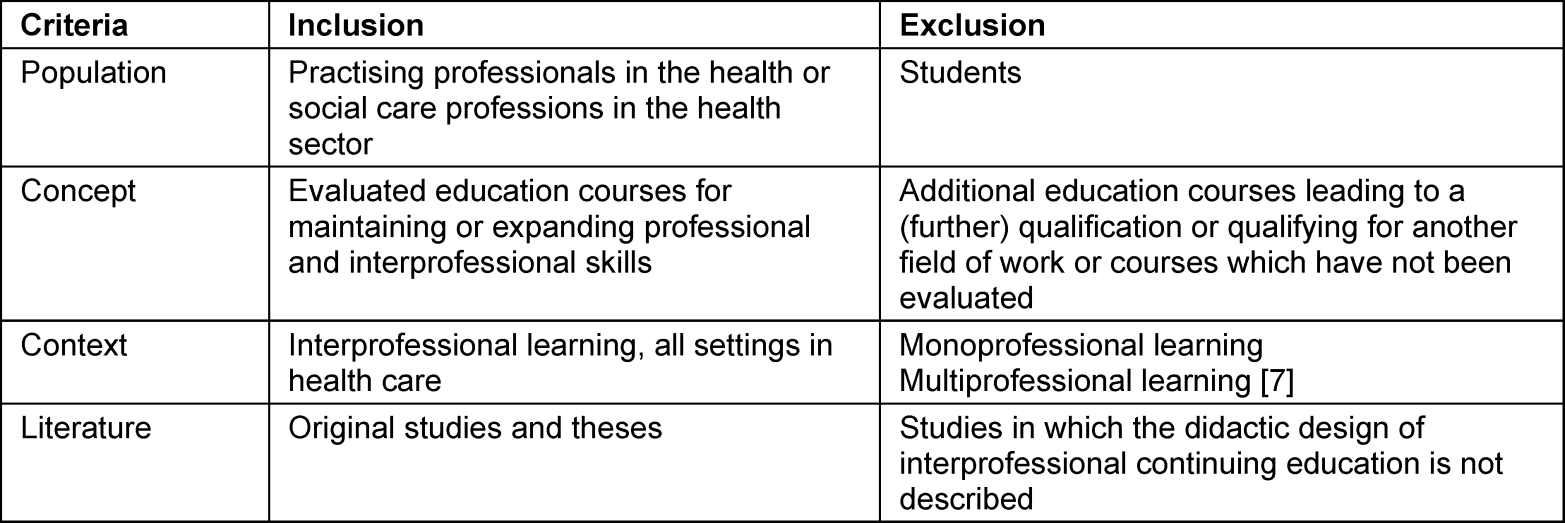
Inclusion and exclusion criteria

**Table 2:**
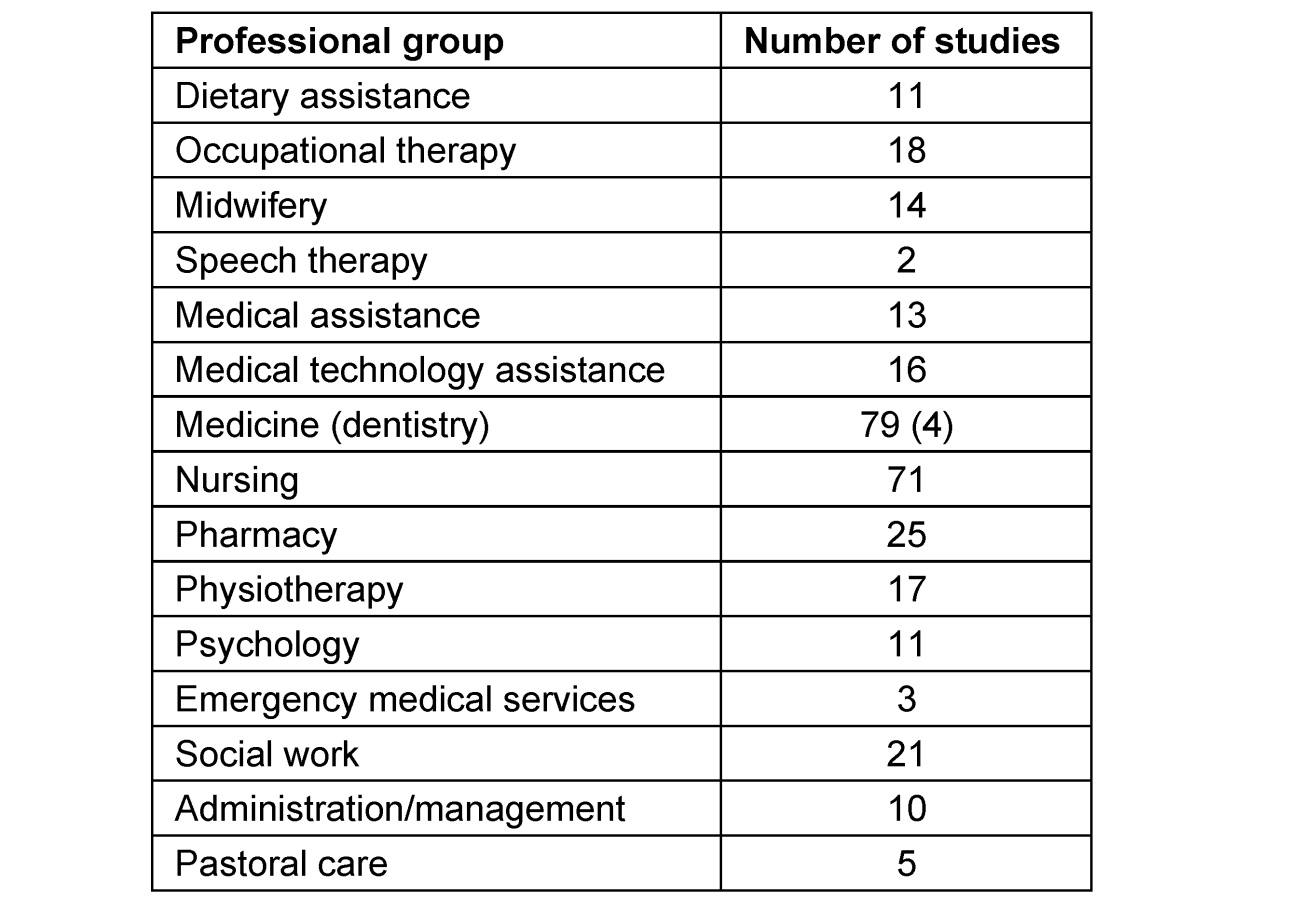
Most common professional groups in continuing interprofessional education
