# Supplementary material for: Interprofessional continuing education in health professions – a scoping review of framework conditions, design processes and evaluation designs
Source: GMS J Med Educ. 2026 Feb 17;43(2):Doc21. doi: 10.3205/zma001815 (PMC12936925; doi:10.3205/zma001815)
Supplement: Search strings in the literature databases [file JME-43-21-s-001.pdf]

Attachment 1: Search strings in the literature databases

MEDLINE (PubMed)

(english[Filter]) OR (german[Filter])

AND

(interprofession*[tiab]) or (inter-profession*[tiab]) or (interdisciplin*[tiab]) or (inter-disciplin*[tiab]) or (interoccupation*[tiab]) or (inter-occupation*[tiab]) or (interinstitut*[tiab]) or (inter-institut*[tiab]) or (intersector*[tiab]) or (inter-sector*[tiab]) or (interdepartment*[tiab]) or (inter-department*[tiab]) or (interprofessional relations/) or (team*[tiab]) or (multiprofessional*[tiab]) or (multi-professional*[tiab]) or (multidisciplin*[tiab]) or (multi-disciplin*[tiab]) or (multiinstitution*[tiab]) or (multi-institution*[tiab]) or (multioccupation*[tiab]) or (multi-occupation*[tiab]) or (multiagenc*[tiab]) or (multi-agenc*[tiab]) or (interagenc*[tiab]) or (inter-agenc*[tiab]) or (multiorganisation*[tiab]) or (multi-organisation*[tiab]) or (multiorganization*[tiab]) or (multi-organization*[tiab]) or (interorganisation*[tiab]) or (inter-organisation*[tiab]) or (interorganization*[tiab]) or (inter-organization*[tiab]) or (exp professional-patient relations/) or (transprofession*[tiab]) or (trans-profession*[tiab]) or (transdisciplin*[tiab]) or (trans-disciplin*[tiab])

AND

(education*[tiab]) or (train*[tiab]) or (learn*[tiab]) or (teach*[tiab]) or (course*[tiab]) or (exp education, continuing/)

AND

(continu*[tiab]) or (postgradua*[tiab]) or (post-gradua*[tiab]) or (ongoing*[tiab]) or (on-going*[tiab]) or (further*[tiab]) or (CIPE[tiab])

AND

(program evaluation/) or (program report/) or (patient outcome*[tiab]) or (teamoutcome*[tiab]) or (team-outcome*[tiab]) or (team outcome*[tiab]) or (education*[tiab] and outcome*[tiab]) or (interdisciplin*[tiab] and outcome*[tiab]) or (inter-disciplin*[tiab] and outcome*)

CINHAL (EBSCO)

(AB interprofession*) or (AB inter-profession*) or (AB interdisciplin*) or (AB inter-disciplin*) or (AB interoccupation*) or (AB inter-occupation*) or (AB interinstitut*) or (AB inter-institut*) or (AB interagen*) or (AB inter-agen*) or (AB intersector*) or (AB inter-sector*) or (AB interdepartment*) or (AB inter-department*) or (AB interorganisation*) or (AB inter-organiza-tion*) or (MH interprofessional relations) or (AB team*) or (AB multiprofession*) or (AB multi-profession*) or (AB multidisciplin*) or (AB multi-disciplin*) or (AB multiinstitution*) or (AB multi-institution*) or (AB multioccupation*) or (AB multi-occupation*) or (AB multiagenc*) or (AB multi-agenc*) or (AB

Attachment 1 to Wesselborg B, Knepperges I, von den Driesch N, Schäfer M, Stephan A. *Interprofessional continuing education in health professions – a scoping review of framework conditions, design processes and evaluation designs.* GMS J Med Educ. 2026;43(2):Doc21. DOI: 10.3205/zma001815

multisector*) or (AB multi-sector*) or (AB multiorganisation*) or (AB multi-organization*) or (MH "Professional-Patient Relations+") or (AB transprofession*) or (AB trans-profession*) or (AB transdisciplin*) or (AB trans-disciplin*) or (TI interprofession*) or (TI inter-profession*) or (TI interdisciplin*) or (TI inter-disciplin*) or (TI interoccupation*) or (TI inter-occupation*) or (TI interinstitut*) or (TI inter-institut*) or (TI interagen*) or (TI inter-agen*) or (TI intersector*) or (TI inter-sector*) or (TI interdepartment*) or (TI inter-department*) or (TI interorganisation*) or (TI inter-organization*) or (TI team*) or (TI multiprofession*) or (TI multi-profession*) or (TI multidisciplin*) or (TI multi-disciplin*) or (TI multiinstitution*) or (TI multi-institution*) or (TI multioccupation*) or (TI multi-occupation*) or (TI multiagenc*) or (TI multi-agenc*) or (TI multisector*) or (TI multi-sector*) or (TI multiorganisation*) or (TI multi-organization*) or (TI transprofession*) or (TI trans-profession*) or (TI transdisciplin*) or (TI trans-disciplin*)

AND

(AB education*) or (AB train*) or (AB learn*) or (AB teach*) or (AB course*) or (TI education*) or (TI train*) or (TI learn*) or (TI teach*) or (TI course*) or (MH "education, continuing+") or (MH "education, graduate+")

AND

(TI continu*) or (TI postgradua*) or (TI post-gradua*) or (TI ongoing*) or (TI on-going*) or (TI further*) or (TI CIPE) or (AB continu*) or (AB postgradua*) or (AB post-gradua*) or (AB on-going*) or (AB on-going*) or (AB further*) or (AB CIPE)

AND

(MH "student performance appraisal+") or (MH "course evaluation") or (MH "program evaluation") or (MH "evaluation research+") or (MH "health care outcome*") or (education* N1 outcome*)

ProQuest „Dissertation & Theses“

(AB(interprofession*) or AB(inter-profession*) or AB(interdisciplin*) or AB(inter-disciplin*) or AB(interoccupation*) or AB(inter-occupation*) or AB(interinstitut*) or AB(inter-institut*) or AB(interagen*) or AB(inter-agen*) or AB(intersector*) or AB(inter-sector*) or AB(interde-partment*) or AB(inter-department*) or AB(interorgani?ation*) or AB(inter-organi?ation*) or AB(team*) or AB(multiprofession*) or AB(multi-profession*) or AB(multidisciplin*) or AB(multi-disciplin*) or AB(multiinstitution*) or AB(multi-institution*) or AB(multioccupation*) orAB(multi-occupation*) or AB(multiagenc*) or AB(multi-agenc*) or AB(multisector*) or AB(multi-sector*) or AB(multiorganisation*) or AB(multi-organization*) or AB(transprofession*) or AB(trans-profession*) or AB(transdisciplin*) or AB(trans-disciplin*) or TI(interprofession*) TI(inter-profession*) or TI(interdisciplin*) or TI(inter-disciplin*) or TI(interoccupation*) or TI(inter-occupation*) or TI(interinstitut*) or TI(inter-institut*) or TI(interagen*) or TI(inter-agen*) or TI(intersec-tor*) or TI(inter-sector*) or TI(interdepartment*) or TI(inter-department*) or TI(interorganisation*) or TI(interorganization*) or TI(team*) or TI(multiprofession*) or TI(multi-profession*) or TI(multidisciplin*) or TI(multi-disciplin*) or TI(multiinstitution*) or TI(multi-institution*) or TI(multioccupation*) or TI(multi-occupation*) or TI(multiagenc*) or TI(multi-agenc*) or

TI(multisector*) or TI(multi-sector*) or TI(multiorganisation*) or TI(multi-organization*) or TI(transprofession*) or TI(trans-profession*) or TI(transdisciplin*) or TI(trans-disciplin*)) AND (AB(education*) or AB(train*) or AB(learn*) or AB(teach*) or AB(course*) or TI(education*) or TI(train*) or TI(learn*) or TI(teach*) or TI(course*)) AND (TI(continu*) or TI(postgradua*) or TI(post-gradua*) or TI(ongoing*) or TI(on-going*) or TI(further*) or TI(CIPE) or AB(continu*) or AB(postgradua*) or AB(post-gradua*) or AB(ongoing*) or AB(on-going*) or AB(further*) or AB(CIPE))

AND

(dissertation or thesis or theses)

PROSPERO

(interprofession* OR inter-profession* OR interdisciplin* OR inter-disciplin* OR interoccupation* OR inter-occupation* OR interinstitut* OR inter-institut* OR intersector* OR inter-sector* OR interdepartment* OR inter-department* OR team* OR multiprofessional* OR multi-profes-sional* OR multidisciplin* OR multi-disciplin* OR multiinstitution* OR multi-institution* OR multioccupation* OR multi-occupation* OR multiagenc* OR multi-agenc* OR interagenc* OR inter-agenc* OR multiorganisation* OR multi-organisation* OR multi-organization* OR multi-organization* OR interorganisation* OR inter-organisation* OR inter-organization* OR inter-organization* OR transprofession* OR trans-profession* OR transdisciplin* OR trans-disciplin*) AND (education* OR train* OR learn* OR teach* OR course*) AND (continu* OR postgradua* OR post-gradua* OR ongoing* OR on-going* OR further* OR CIPE) AND (program evaluation/ OR program report/ OR patient outcome* OR teamoutcome* OR team-outcome* OR team outcome* OR (education* and outcome*) OR (interdisciplin* and outcome*) OR (inter-disciplin* and outcome*))

Search fields: Titel, Final Report, Review Question, Searches, Summary
